# Emotional Abuse and Psychological Distress in Individuals with Multiple Sclerosis: The Mediation Role of Internalized Shame and Chronic Illness Stigma

**DOI:** 10.3390/bs16050632

**Published:** 2026-04-23

**Authors:** Nadia Barberis, Giorgio Falgares, Giulia Costanzo, Marco Cannavò

**Affiliations:** 1 Dipartimento di Scienze Teoriche e Applicate, Università degli Studi eCampus, 22060 Novedrate, Italy; nadia.barberis@uniecampus.it; 2 Dipartimento di Scienze Psicologiche, Pedagogiche, dell’Esercizio Fisico e della Formazione, Università degli Studi di Palermo, 90133 Palermo, Italy; giorgio.falgares@unipa.it; 3 Dipartimento di Scienze della Salute, Università degli Studi Magna Graecia di Catanzaro, 88100 Catanzaro, Italy; marco.cannavo@unicz.it

**Keywords:** emotional abuse, internalized shame, stigma, chronic illness, distress, depression, anxiety, stress, multiple sclerosis

## Abstract

Multiple sclerosis (MS) is a debilitating neurological condition that affects several domains of individuals’ lives, making those affected particularly vulnerable to psychological distress. The visible nature of many MS symptoms may increase self-consciousness, thereby fostering feelings of shame and perceived stigma. Previous research has shown that self-related perceptions are shaped by early interpersonal relationships, rendering emotional trauma particularly relevant in this context. The present study sought to test whether an association between emotional abuse and psychological distress (Depression, Anxiety, and Stress) in individuals with MS would be mediated by internalized shame and perceived stigma. A total of 171 individuals with a clinical diagnosis of MS (85% women; M = 30.04, SD = 10.01) were recruited and completed a set of validated questionnaires assessing the variables of interest. Structural Equation Modeling was used to test the proposed model. Significant paths emerged from emotional abuse to internalized shame and from emotional abuse to internalized shame. In addition, internalized shame was significantly associated with psychological distress, and a further significant path was observed from perceived stigma to psychological distress. Moreover, significant indirect effects were found from emotional abuse to psychological distress via internalized shame and via perceived stigma.

## 1. Introduction

### 1.1. Multiple Sclerosis: Clinical Features and Disease Burden

Multiple sclerosis (MS) is a chronic, immune-mediated disease of the central nervous system characterized by inflammation, demyelination, and neurodegeneration. Its pathology involves an aberrant immune response targeting myelin and axons in the brain and spinal cord, resulting in focal lesions and diffused tissue damage. Clinically, MS is marked by substantial heterogeneity in disease course, symptom presentation, and levels of impairment. Symptoms vary in severity, combination, and temporal evolution across individuals and commonly include sensory disturbances (such as numbness, paresthesia, and dysesthesia), visual impairment, and motor dysfunction manifested by weakness, spasticity, and gait instability. Additional features frequently observed include cognitive impairment affecting attention, processing speed, and memory, as well as bladder and bowel dysfunction, sexual dysfunction, pain syndromes, and fatigue, which is often disproportionate to physical activity (Dobson & Giovannoni, 2018; Montalban et al., 2025). Recent epidemiological evidence highlights the growing global burden of MS. In 2021, an estimated 1.89 million people worldwide were living with MS, with over 62,000 new diagnoses and a global prevalence of 23.9 cases per 100,000 population, steadily rising over the past three decades (Khan & Hashim, 2025). The chronic and disabling nature of MS imposes significant challenges on individuals, families, and society. A cross-sectional study across 16 European countries reported that, as disease severity increases, work capacity falls sharply from 82% to 8%, while health-related utility declines from population norms to below zero, underscoring employment loss as a major contributor to both societal and individual economic burden (Kobelt et al., 2017). Consistently, the employment gap between people with MS and the general population ranges from 15% to 20% (Kavaliunas et al., 2021).

### 1.2. Psychological Distress in Multiple Sclerosis

In addition to MS-related pain, individuals with multiple sclerosis frequently experience psychological distress arising from the multifaceted nature of the condition (Fisher et al., 2020; Meek et al., 2022), which further complicates clinical management. Psychological distress is commonly defined as a multidimensional construct encompassing symptoms of depression, anxiety, and stress, including hopelessness, perceived threat, irritability, and frustration, that emerge in response to internal or external stressors (Makara-Studzińska et al., 2022; Varela et al., 2017). Robust evidence indicates that affective symptoms are highly prevalent in MS. A recent systematic review and meta-analysis carried out by Peres et al. (2022) reported elevated prevalence rates of both depression and anxiety across all clinical forms of MS, with higher rates observed in progressive forms and a clear association with greater impairment. Similarly, findings from the study performed by Young et al. (2024) emphasized that depression in MS is not only common but also frequently under-treated, and is strongly linked with fatigue, pain, and worsened quality of life. Furthermore, stress has been identified as a critical factor influencing disease course: recent meta-analytic findings provided by von Drathen et al. (2024) highlighted that psychological stress is linked with greater risk of relapse, may contribute to disease onset, and could influence long-term impairment progression, highlighting stress as a clinically relevant and potentially modifiable factor. These findings highlight the critical role of psychological comorbidities in shaping clinical outcomes and the importance of systematically assessing and managing mental health in MS care. This is especially relevant given projections that the global prevalence of MS is expected to rise in the coming decades, underscoring the growing public health significance of the disease and the need for context-specific strategies for prevention, surveillance, and healthcare planning (Khan & Hashim, 2025). Because MS often involves visible symptoms, such as gait impairment, balance difficulties, or the use of mobility aids, individuals may become concerned about their appearance and how others perceive it. As a result, people with MS may experience negative attitudes, social devaluation, and discriminatory behaviors directed toward them due to their disease or its manifestations—commonly referred to as illness stigma (Pescosolido & Martin, 2015).

### 1.3. Illness Stigma in Multiple Sclerosis

Illness stigma is conceptualized as a feeling of discrimination experienced by individuals because of a health condition. More specifically, a range of studies has shown that endorsing negative beliefs and feelings related to a stigmatized attribute is common in neurological conditions (Elliot et al., 2019; Rao et al., 2009). In the context of MS, a recent systematic review (Powell et al., 2024) found that stigma is associated with poorer psychological well-being, higher levels of depression and anxiety, and reduced psychosocial adjustment, underscoring its significant impact on both psychosocial functioning and overall health outcomes. Consistently, a correlational study by Sharifi et al. (2023) reported that greater perceived stigmatization was linked to lower quality of life across physical, emotional, cognitive, social, and sexual domains. Factors such as longer disease duration, role limitations, pain, and poorer health perception further negatively affected quality of life, whereas marriage, better physical health, greater emotional well-being, and lower health distress were positively associated.

### 1.4. Internalized Shame in Multiple Sclerosis

A key aspect of stigmatization lies in its relational origin and its grounding in processes of negative self-evaluation (Earnshaw & Quinn, 2011). Accordingly, it is important to further examine how stigma may interact with factors arising from actual or anticipated experiences of social devaluation, rejection, or discrimination, particularly in the context of chronic illness. In this regard, empirical research highlights the role of specific emotions in fostering an unhealthy preoccupation with social evaluation and feelings of defectiveness (Barta & Kiropoulos, 2023; Dolezal, 2022; Schmader & Lickel, 2006). Building on this perspective, a growing body of evidence suggests that self-conscious emotions, such as shame, may critically influence individuals’ awareness or expectation of negative social judgments. Shame is a painful self-conscious emotion marked by feelings of personal inadequacy and diminished self-worth (Tracy & Robins, 2004), which frequently motivates avoidance, concealment, or social withdrawal in response to perceived failures or threats (Tangney et al., 2007; Tignor & Colvin, 2019). Internalized shame, as a core dimension of this emotional experience, reflects the incorporation of negative self-judgments into one’s self-concept and is strongly associated with maladaptive self-referential cognitions and affective processes (Gilbert, 2000). In this regard, cross-sectional evidence provided by Barta and Kiropoulos (2023) elucidated the psychological mechanisms linking depression and anxiety to attitudes toward psychological help-seeking in people with MS. Using a mediation model, they demonstrated that internalized shame and stigma play a central mediating role, such that higher levels of depression and anxiety are associated with greater shame and perceived stigma, which in turn predict more negative attitudes toward seeking psychological support. These findings are further supported by the systematic review by Powell et al. (2024), which included 18 studies and found that stigma was significantly associated with adverse psychological and physical health outcomes. It is thought that experiences of shame arise from repeated social experiences perceived as humiliating or devaluing (Klaassen, 2001; Tangney et al., 2007). Accordingly, interpersonal interactions characterized by invalidation, verbal aggression, and humiliation are likely to foster shame-related processes (Karan et al., 2014; Dorahy et al., 2016). It is, therefore, important to examine the impact of such maladaptive interpersonal dynamics on the development and modulation of internalized shame.

### 1.5. Implications of Emotional Abuse

In this regard, the concept of emotional abuse effectively captures patterns of verbal hostility, humiliation, and emotional neglect that can erode individuals’ confidence and self-worth (Dorahy, 2010; Shi, 2013). Shame has been identified as a common emotional consequence of various forms of interpersonal trauma, including physical and sexual victimization, intimate partner violence, childhood maltreatment, and racial discrimination (Badour et al., 2017; Beck et al., 2011; DeCou et al., 2019; Matheson & Anisman, 2009; Sekowski et al., 2020). Among adverse childhood experiences, however, emotional abuse and neglect appear to exert a particularly strong and enduring influence on the development of shame. Due to its chronic and relational nature, emotional abuse is consistently associated with repeated exposure to demeaning evaluations during childhood, which tend to be internalized over time.

In addition to direct forms of demeaning evaluation, indirect relational experiences such as repeated comparison with peers by caregivers may further contribute to the development of negative self-perceptions. When children are consistently compared unfavorably to others, they may internalize a sense of inferiority, inadequacy, and conditional self-worth, reinforcing beliefs of being defective or not good enough (Gilbert, 2000; Parker, 1993). Such experiences may function as chronic social-evaluative stressors, increasing sensitivity to external judgment and fostering maladaptive self-referential processing. Over time, these patterns may contribute to the development of internalized shame and heightened vulnerability to perceived stigma (Major & O’Brien, 2005; Tangney & Dearing, 2002).

### 1.6. Study Rationale

Higher levels of interpersonal hostility, denigration, or rejection have been linked to a greater tendency for negative external appraisals to be incorporated into self-evaluation processes, a pattern commonly observed into shame-related processes (Zhang et al., 2025). The hostility, insults, and aggression expressed in emotionally traumatizing caregiver behaviors are associated with the development of negative self-evaluations in abused children, including perceptions of being defective, defiled, or unworthy (Howell, 2014; Sekowski et al., 2020; Kaufman & Zigler, 1996). Such negative self-views are closely linked to feelings of unworthiness and defectiveness, which constitute core features of shame-related experiences (Passanisi et al., 2015). Within this framework, a stable and enduring form of shame—namely internalized shame—may emerge, whereby negative external evaluations, criticisms, or demeaning interpersonal experiences are incorporated into the self-concept, leading individuals to perceive themselves as fundamentally flawed or unworthy. In addition, emotional abuse during childhood, particularly in the form of exposure to critical, hostile, or dismissive caregiving, is closely linked to interpersonal experiences characterized by devaluation, rejection, and invalidation, which may shape enduring expectations about how one is perceived by others (Dorahy, 2010; Gilbert, 2000). Repeated experiences of emotional abuse or emotional neglect have been linked to the development of negative self-schemas and heightened sensitivity to social evaluation, patterns that may persist into adulthood (Kaufman & Zigler, 1996; Sekowski et al., 2020).

Furthermore, these early experiences of emotional abuse and neglect may have long-term implications for interpersonal relationships and internal self-concepts in adulthood. Individuals exposed to chronic criticism, hostility, or emotional invalidation during childhood are more likely to develop insecure interpersonal patterns, heightened sensitivity to rejection, and enduring beliefs of inadequacy or unworthiness. These maladaptive interpersonal representations and internalized self-views may persist over time, shaping how individuals interpret and respond to interpersonal interactions in adulthood (Gilbert, 2000; Mikulincer & Shaver, 2016). In the context of chronic conditions such as MS, these vulnerabilities may become particularly salient. The challenges associated with MS, including symptom visibility, functional limitations, and social misconceptions, may activate pre-existing sensitivities to evaluation and rejection, thus exacerbating internalized shame, perceived stigma, and psychological distress (Phelan et al., 2008; Major & O’Brien, 2005; Earnshaw & Quinn, 2011).

**Figure 1:**
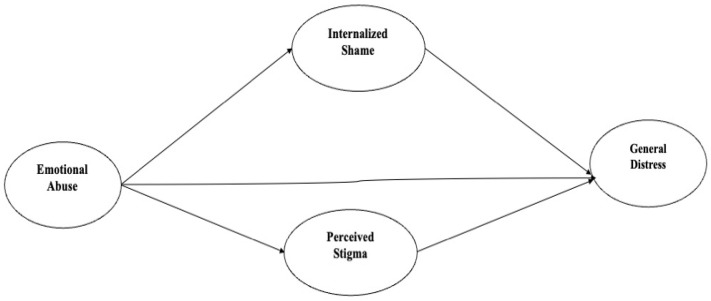
Hypothesized model.

Consequently, individuals with a history of emotional abuse may be more likely to anticipate judgment, exclusion, or social devaluation in interpersonal contexts, thereby reporting higher levels of perceived stigma (Earnshaw & Quinn, 2011). In the context of chronic illness, such as MS, these vulnerability factors may interact with illness-related visibility and social stereotypes, amplifying perceptions of being negatively evaluated or discriminated against because of the condition (Powell et al., 2024). Individuals with MS often contend with both visible symptoms, such as gait impairment, tremors, or the use of mobility aids, and invisible symptoms, including fatigue, cognitive difficulties, and chronic pain. Misunderstandings surrounding these symptoms, together with the unpredictable disease course and prevailing societal stereotypes related to dependence, are frequently associated with experiences of discrimination, social avoidance, and negative judgments, thereby increasing the likelihood of heightened psychological distress. Drawing from these theoretical and practical insights, the present study aimed to test a mediation model in which the association between emotional abuse and psychological distress (i.e., depression, anxiety, and stress) in individuals with multiple sclerosis is mediated by internalized shame and perceived stigma. More specifically, it was hypothesized that higher levels of emotional abuse would be associated with higher levels of internalized shame and perceived stigma, which in turn would be associated with higher levels of psychological distress. Additionally, a direct positive association between emotional abuse and psychological distress was expected (Figure 1).

## 2. Materials and Methods

### 2.1. Method and Design

The present study used a cross-sectional design to observe the relationship between the variables of interest. More specifically, a Structural Equation Modelling (SEM) with latent variables approach was used. Figure 1 depicts the hypothesized model where internalized shame and perceived stigma are parallel mediators in the relationship between emotional abuse and psychological distress. Data were gathered online via social network dissemination.

### 2.2. Participants

Participants were 171 individuals (85% women) with a clinical diagnosis of MS (M = 30.04; SD = 10.01). All participants identified as Caucasian and were native Italian speakers. Demographics and sample characteristics can be found in Table 1.

### 2.3. Measures

#### 2.3.1. Emotional Abuse

Emotional abuse was assessed using the Emotional Abuse subscale of the Childhood Trauma Questionnaire–Short Form (CTQ-SF; Bernstein et al., 1998) in its Italian validation (Sacchi et al., 2018). This self-report questionnaire consists of 5 items and assesses the degree to which individuals experienced emotional abuse during childhood. (e.g., “When I was growing up, people in my family said hurtful or insulting things to me”), rated on a 5-point Likert scale (1 = “Never true” to 5 = “Very often true”). The CTQ-SF shows robust psychometric properties, including structural validity, measurement invariance, and reliability (Badenes-Ribera et al., 2024; Georgieva et al., 2021). Internal consistency in the current study is reported in Table 2.

#### 2.3.2. Internalized Shame

The Internalized Shame Scale (Cook, 1988, 1994) is a self-report questionnaire that consists of 35 items and assesses shame-related self-evaluations (e.g., “I feel like I am never quite good enough”), rated from 0 (“Never”) to 4 (“Always”). It is widely used in research on internalized shame (Lear et al., 2022). Internal consistency for the present study is reported in Table 2.

#### 2.3.3. Perceived Stigma

Perceived stigma was measured using the Stigma Scale for Chronic Illnesses (Molina et al., 2013). It is a self-report scale that consists of 8 items and assesses negative social attributions related to illness (e.g., “Lately, because of my illness, some people seemed uncomfortable with me”), rated from 1 (“Never”) to 5 (“Very often true”). It has been validated in neurological populations (Hayat et al., 2025; Yoo et al., 2017) and demonstrates strong psychometric properties (Sanabria-Mazo et al., 2025). Internal consistency in the current study is reported in Table 2.

#### 2.3.4. Psychological Distress

Psychological distress was assessed with the Depression, Anxiety, and Stress Scale–21 (DASS-21; Lovibond & Lovibond, 1995) in its Italian validation (Bottesi et al., 2015). This scale consists of 21 items and comprises three subscales—anxiety, depression, and stress (7 items each)—with items rated from 0 (“Did not apply to me at all”) to 3 (“Applied to me very much or most of the time”). The DASS-21 is widely used in populations with medical conditions (Barberis et al., 2023a, 2023b, 2023c) and exhibits strong psychometric properties (Vignola & Tucci, 2014; Zanon et al., 2020). Internal consistency for this study is reported in Table 2.

### 2.4. Procedures

Participants with MS were recruited through targeted social media advertisements posted in thematic groups. Inclusion criteria required a physician-confirmed diagnosis of MS for at least one year, age of 18 years or older, and Italian as a first language. Individuals were excluded if they had neurological comorbidities, were younger than 18 years, or were not native Italian speakers. To minimize missing data, all advertisements directed respondents to an anonymous online survey with mandatory response fields. Prior to participation, individuals were informed of the voluntary nature of the study and provided electronic informed consent. Data were analyzed using SPSS 27 (IBM Corp., 2023) and R 4.3.0 (R Core Team, 2023), with the integration of RStudio 2023.06.0 (RStudio Team, 2023) and the Lavaan package (v0.6-x; Rosseel, 2012). The study was approved by the Ethical Committee of the Center for Research and Psychological Intervention at the University of Messina and was conducted in accordance with the Ethical Code of the Italian Association of Psychology and the 1964 Helsinki Declaration, including its subsequent amendments.

### 2.5. Statistical Analyses

**Figure 2:**
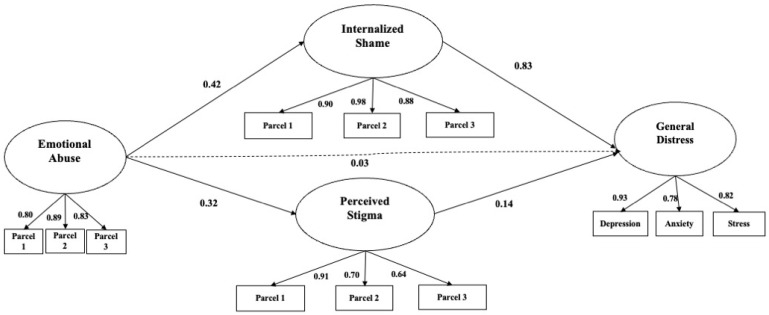
Model representing the relationships between study variables. Note: Circles represent the latent variables, boxes represent the observed variables. The numerical values on the arrows between latent variables are standardized multiple regression coefficients. The table depicts only significant paths for the sake of clarity.

In a first step, descriptive analyses and Pearson correlations were conducted for all observed variables. In a second step, structural equation modeling (SEM) with latent variables was used to examine the relationships among the observed variables (Figure 2). Specifically, a structural equation model with latent variables was specified to test a model in which emotional abuse served as the predictor variable, internalized shame and perceived stigma as mediators, and psychological distress as the outcome variable. For the psychological distress latent construct, the three scales assessing anxiety, depression, and stress were used. For all other latent constructs, a parceling approach was adopted, consisting of the aggregation of randomly selected items from each scale into three indicators per latent variable (Little et al., 2002). Parcels are less likely to be influenced by method effects and are more likely to meet the assumption of normality (Little et al., 2002; Marsh et al., 1998). The analysis was conducted on the covariance matrices, and model solutions were estimated using maximum likelihood estimation.

## 3. Results

### 3.1. Descriptives and Correlations

Means, standard deviations, skewness, and kurtosis for each variable are reported in Table 2. Additionally, Table 2 presents the correlations among the observed variables and the internal consistency indices for each questionnaire.

### 3.2. Mediation Model

The model demonstrated acceptable fit indices: χ^2^(49) = 89.81, *p* < 0.001; CFI = 0.97; RMSEA = 0.07 (90% CI [0.05, 0.09]); and SRMR = 0.08 (Figure 2). Significant paths were observed from emotional abuse to internalized shame (β = 0.42) and from emotional abuse to internalized shame (β = 0.32). In addition, significant paths were found from internalized shame to psychological distress (β = 0.81). A further significant path emerged from perceived stigma to psychological distress (β = 0.14). Moreover, a significant indirect effect was identified from emotional abuse to psychological distress via internalized shame (β = 0.34). An additional indirect effect was observed from emotional abuse to psychological distress via perceived stigma (β = 0.05). A detailed description of the path estimates is provided in Table 3.

## 4. Discussion

The present study aimed to examine whether the association between emotional abuse and psychological distress (depression, anxiety, and stress) in individuals with MS is mediated by internalized shame and perceived stigma. The findings support the hypothesized mediational model, highlighting the role of both intrapersonal and interpersonal processes in explaining this relationship. Specifically, internalized shame and perceived stigma emerged as significant mediators, suggesting that the psychological impact of emotional abuse operates primarily via alterations in self-concept and social perception rather than through a direct effect on distress.

These results are parallel with theoretical and empirical frameworks positing that early experiences of emotional abuse and neglect foster the development of negative self-schemas, increased sensitivity to social scrutiny, and maladaptive self-referential processes (Gilbert, 2000; Parker, 1993; Tangney & Dearing, 2002). Internalized shame, reflecting the incorporation of negative self-evaluations into one’s identity, appears to represent a pivotal mechanism linking adverse relational experiences to vulnerability to internalizing symptoms. Similarly, perceived stigma captures the socially mediated dimension of this process, whereby expectations of devaluation and discrimination foster greater psychological distress. Overall, the findings align with prior research in MS populations (Powell et al., 2024; Sharifi et al., 2023), highlighting the importance of the interplay between intrapersonal and interpersonal factors in psychological maladjustment.

More specifically, internalized shame was found to mediate the relationship between emotional abuse and psychological distress. Exposure to critical and hostile interpersonal environments during early developmental stages fosters heightened sensitivity to social evaluation (Gilbert, 2000; Kaufman & Zigler, 1996), fostering persistent feelings of defectiveness and unworthiness. In line with previous studies on chronic conditions (Cannavò et al., 2025b; Trindade et al., 2017, 2018), such negative self-schemas may contribute to avoidance of potentially evaluative situations and to heightened vulnerability to internalizing distress. Moreover, emotionally invalidating caregiving contexts, characterized by dismissal of the child’s perspective and limited opportunities for emotional expression, may foster withdrawal as a strategy to regulate distress and may impair the development of mentalizing processes (Allen et al., 2008; Fonagy & Luyten, 2009; Fonagy & Bateman, 2016). These processes may, in turn, increase proneness to shame and difficulties in interpreting social cues. Consistent with prior cross-sectional evidence in MS populations (Meek et al., 2022; Pakenham et al., 2023), higher levels of internalized shame were linked with heightened psychological distress, supporting the role of a negatively biased self-concept in maintaining emotional suffering.

Contrary to expectations, the relationship between emotional abuse and psychological distress was not statistically significant. This finding may indicate that the impact of emotional abuse on distress operates primarily via internalized shame and perceived stigma. In other words, the psychological consequences of emotional abuse may not manifest uniformly but rather through intrapersonal and interpersonal processes that shape self-concept and social perception. Such a pattern aligns with theoretical perspectives suggesting that internalized self-judgments and socially mediated perceptions are central mechanisms linking early maladaptive relationships to later emotional maladjustment (Gilbert, 2000; Tangney & Dearing, 2002). These results, hence, highlight the importance of examining mediational pathways, rather than relying solely on direct associations, to fully understand the psychological sequelae of emotional abuse in chronic conditions such as MS.

Perceived stigma also emerged as a significant mediator, indicating that emotionally abusive experiences may foster enduring expectations of social rejection or discrimination. These expectations may heighten sensitivity to negative social evaluation, leading individuals to interpret interpersonal interactions through a stigmatizing lens (Powell et al., 2024). In the context of MS, such perceptions may be further reinforced by visible symptoms (e.g., gait impairment, balance difficulties, use of mobility aids), which can amplify concerns about social judgment (Sharifi et al., 2023). This process may promote hypervigilance to social threat, reinforce maladaptive beliefs about the self and others, and contribute to both anxiety- and stress-related responses, as well as depressive symptoms characterized by social withdrawal and loneliness. These findings are consistent with previous reviews highlighting the central role of stigma in MS (Powell et al., 2024) and the interplay between emotional abuse and affective disturbances in this population (Polick et al., 2022).

Several limitations should be acknowledged. The cross-sectional design precludes causal inferences, and future longitudinal or experimental studies are needed to test the proposed model more rigorously. The exclusive reliance on self-report measures may have introduced bias; thus, future research should incorporate multi-method assessments, including clinical interviews. Additionally, self-selection bias may have influenced the sample composition, potentially overrepresenting individuals with higher distress levels. Finally, the sample was limited to Italian Caucasian participants, restricting the generalizability of the findings. Replication in more diverse socio-cultural contexts is warranted.

Despite these limitations, the present study offers relevant theoretical and clinical implications, emphasizing the importance of targeting internalized shame and perceived stigma in interventions aimed at reducing psychological distress in individuals with MS.

The present findings highlight the central role of internalized shame and perceived stigma as key psychological mechanisms linking emotional abuse to psychological distress in individuals with MS. These results underscore the importance of systematically addressing self-conscious emotions—particularly internalized shame—as well as socially mediated perceptions such as perceived stigma in both clinical assessment and intervention. From an assessment perspective, this implies the need to move beyond the exclusive evaluation of general distress symptoms to include more nuanced dimensions related to self-evaluation and interpersonal sensitivity. From an intervention standpoint, these findings suggest that targeting maladaptive self-representations, fear of negative evaluation, and stigma-related beliefs may be particularly relevant for reducing vulnerability to internalizing symptoms and promoting more adaptive psychological adjustment. Targeting these processes may contribute to improving overall psychological well-being in this population. Current results also suggest that individuals with MS may be particularly susceptible to internalizing difficulties. Clinicians should routinely assess not only anxiety, depression, and stress but also self-conscious emotions, particularly internalized shame, to improve treatment effectiveness. This is particularly relevant, given that an emerging number of insights from the research in health psychology is warning that management of the emotional profile is key for avoiding complications in chronic conditions (Cannavò et al., 2024, 2025a). From a public health perspective, the results highlight the harmful effects of perceived stigma in this population and underscore the need for targeted interventions to promote health literacy and awareness of the psychosocial impact of MS (Bullivant et al., 2020). Effective programs should engage not only patients but also socialization agents such as family, peers, and the media to foster understanding and reduce stigma.

## 5. Conclusions

The present study advances understanding of psychological distress in multiple sclerosis by highlighting the central role of self-conscious and socially mediated processes. Rather than supporting a direct link between emotional abuse and distress, the findings indicate that this association operates primarily through internalized shame and perceived stigma, underscoring how adverse relational experiences are processed and incorporated into the self-concept. Within chronic illness, these mechanisms appear particularly salient, reflecting the interplay between illness visibility, social evaluation, and pre-existing relational vulnerabilities. This perspective shifts the focus from exposure to adverse experiences per se to their ongoing impact on self-perception and social expectations. Clinically, the findings point to the need for interventions that directly target shame-related processes and stigma sensitivity. This requires moving beyond symptom-focused approaches toward strategies aimed at modifying maladaptive self-representations, reducing fear of negative evaluation, and improving interpersonal functioning. Overall, this study offers a process-oriented framework for understanding distress in multiple sclerosis, emphasizing the role of intrapersonal and interpersonal mechanisms in shaping psychological vulnerability and adjustment.

## Figures and Tables

**Table 1:** Demographics and sample characteristics.

Variable	Category	n	%
Gender	Women	145	85
	Men	26	15
Education Level	Middle school	15	9
	High school diploma	74	43
	University degree	79	46
	Postgraduate degree	3	2
Employment Status	Employed	99	58
	Freelancers	22	13
	Students	12	7
	Unemployed	12	7
	Homemakers	9	5
	Retired	3	2
Marital Status	Single	43	25
	Married	43	25
	Cohabiting	31	18
	Engaged	31	18
	Widowed	14	8
	Divorced	10	6

**Table 2:** Descriptive analysis and correlations.

	α	M	SD	Skew	Kurt	1	2	3	4	5
1. Depression	0.92	1.31	0.80	0.49	−0.80					
2. Anxiety	0.85	1.38	0.72	0.42	−0.51	0.72 **				
3. Stress	0.87	1.80	0.65	−0.04	−0.58	0.76 **	0.76 **			
4. Perceived Stigma	0.84	1.74	0.68	1.16	1.15	0.36 **	0.37 **	0.29 **		
5. Internalized Shame	0.98	1.51	0.97	0.47	−0.67	0.81 **	0.60 **	0.68 **	0.32 **	
6. Emotional Abuse	0.85	1.72	0.83	1.50	2.56	0.39 **	0.29 **	0.29 **	0.30 **	0.39 **

Note: *N* = 171; ** *p* < 0.01.

**Table 3:** Path Estimates, SEs and 95% CIs.

	β	*p*	SE	Lower Bound (BC) 95% CI	Upper Bound (BC) 95% CI
Direct Effect					
Emotional Abuse→Internalized Shame	0.42	<0.001	0.61	0.38	0.84
Emotional Abuse→Perceived Stigma	0.32	<0.001	0.39	0.18	0.60
Emotional Abuse→Psychological Distress	0.03	0.57	0.04	−0.09	0.16
Internalized Shame→Psychological Distress	0.81	<0.001	0.61	0.52	0.71
Perceived Stigma→Psychological Distress	0.14	<0.05	0.13	0.22	0.14
Indirect Effect via Internalized Shame					
Emotional Abuse→Psychological Distress	0.34	<0.001	0.37	0.22	0.52
Indirect Effect via Perceived Stigma					
Emotional Abuse→Psychological Distress	0.05	<0.05	0.05	0.00	0.10

Note: *p* = level of significance; SE = Standards Errors; BC 95% CI = Bias Corrected-Confidence Interval.

## Data Availability

The data presented in this study are available on request from the corresponding author due to the sensitive nature of the clinical health-related information collected and the need to protect participants’ privacy in accordance with ethical and institutional regulations.
